# Linking Databases in Collaborative and Culturally Safe Ways to Evaluate the Effectiveness of PAX-Good Behaviour Game (PAX) in First Nations Communities.

**DOI:** 10.23889/ijpds.v7i3.1961

**Published:** 2022-08-25

**Authors:** Mariette Chartier, Garry Munro, Nora Murdock, Frank Turner, Leanne Boyd, Laurence Katz, Marni Brownell, Depeng Jiang, Amanda Martinson, Janique Fortier, Jitender Sareen

**Affiliations:** 1 Manitoba Centre for Health Policy, University of Manitoba; 2 Cree Nation Tribal Health; 3 Manitoba First Nations Education Resource Centre; 4 Manitoba Adolescent Treatment Centre; 5 University of Manitoba; 6 Government of Manitoba

## Objectives

The overarching research objective was to examine, culturally adapt, and further evaluate a mental health promotion approach called the PAX within 8 First Nations communities. This presentation describes a research process whereby First Nations community members and researchers worked in collaborative and culturally safe ways to reach their research objectives.

## Approach

Building on a strong existing relationship between Swampy Cree Tribal Council (SCTC) members from Northern Canada and academic researchers, a team was formed to prepare the research proposal. This team included community members, leaders from First Nations organizations, decision makers, program developers and researchers. This research was guided by two-eyed seeing, a principle developed by a Mi’kmaw Elder, that recognizes both Indigenous and Western ways of knowing, where one worldview does not dominate the other. The research process was compliant with Ownership, Control, Access, and Possession (OCAP) principles that ensure self-determination of First Nations communities over research involving their people.

## Results

A First Nations community liaison was hired as a research team member ensuring that traditional and cultural protocols were adhered to and connections to community members facilitated and sustained. Over the course of the research, the team met monthly to oversee implementation and annually with SCTC community members for guidance and for sharing and interpreting results. All 8 communities were actively engaged and benefitted from their involvement. Seeing the value of examining PAX’s effectiveness through linkages to administrative datasets, community members supported engagement of an additional 16 First Nations communities thereby ensuring an adequate sample size for the study. Health and education databases were linked to program data from 20 First Nations communities. Infographics, lay summaries and presentations were prepared for meaningful knowledge exchange.

## Conclusion

First Nations communities deemed it essential to understand what works and for whom regarding mental health promotion. Building relationships with First Nations community members based on trust and respect provided information that was relevant and beneficial to their communities. This relationship-building should be considered when developing research timelines and budgets.

